# Readmissions in the Emergency Department

**DOI:** 10.1186/1757-7241-20-S2-O4

**Published:** 2012-04-16

**Authors:** Mads Strunk Mouridsen, Jakob Lundager Forberg, Charlotte Barfod

**Affiliations:** 1 RN, Department of Emergency Medicine, Hillerød Hospital, Denmark; 2 MD, Department of Emergency Medicine, Hillerød Hospital, Denmark; 3 MD, Department of Anaesthesiology, Hillerød Hospital, Denmark

## Background

Overcrowding in the Emergency Department (ED) is a common problem. Patients with frequent admissions within a limited period of time may represent a group of patients, where readmissions could be prevented. However, these patients are not well recognised and the potential benefit of a specific effort to reduce the number of readmissions is unknown.

## Methods

All patients admitted to the ED at Hilleroed Hospital were registered during a 6-months period from September 2009 to March 2010. The ED has approximately 50,000 patient contacts annually, a level 2 Trauma centre and the following specialties: Internal Medicine, Neurology, Surgery and Orthopaedics. Paediatric patients (medicine) and patients to Obstetrics/Gynaecology are admitted directly to the general ward and not through the ED. All contacts resulting in admission were retrieved.

## Results

A total of 20,408 patient contacts were registered in the ED during this period. 7,588 contacts were admitted from the ED. 32 patients had frequent re-admissions representing 198 contacts. The number of readmissions varied from 5 to 15.

In the primary triage 2.6% of the frequently admitted patients, were categorized red (most urgent), 24.1% were orange, 45% were yellow, and 28.3% were green (least urgent). The most common complaints were abdominal complaints (30.4%) chest pain (14.7%) and dyspnoea (14.7%). The median length of stay in the ED for all groups were 13 hours, for contacts with abdominal complaints, 14 hours, chest pain 1.5 hours and dyspnoea 11 hours. For contacts with abdominal complaints, 47% were admitted to a general ward and 53% were discharged from the ED. For chest pain contacts 89% were admitted and 11% discharged, and for contacts with dyspnoea 68% were admitted and 32% discharged.

## Conclusion

Patients readmitted 5 times or more, within a 6-month period most often presents with abdominal complaints. These patients also had the longest stay within the ED.

An increased focus on this group of patients with abdominal complaints could help avoid the need for later readmissions and could help improve ambulatory care for these patients and potentially reduce overcrowding in the ED.

